# Presumed Simultaneous Intraocular and Extraocular BCG-osis as a Complication of Intravesical Bacillus Calmette-Guérin Treatment for Non-Muscle-Invasive Bladder Cancer: A Case Report

**DOI:** 10.7759/cureus.114020

**Published:** 2026-08-05

**Authors:** Natasha S Goh

**Affiliations:** 1 Ophthalmology, Townsville Hospital, Townsville, AUS

**Keywords:** bacillus calmette-guérin (bcg) immunotherapy, disseminated bcg-osis, disseminated tb, turbt, uveitis

## Abstract

Bacillus Calmette-Guérin (BCG) is a live attenuated vaccine that is used as an adjunct following transurethral resection of bladder tumour (TURBT). This treatment is highly effective at reducing the recurrence and progression of the disease, but has a risk of disseminated tuberculosis (TB) infection. Rarely, this can result in ocular involvement, which can manifest as an extraocular granulomatous lesion external to the eye, or intraocular inflammation. This case report presents the simultaneous manifestations of both intraocular and extraocular complications in a 64-year-old gentleman with disseminated TB after receiving BCG immunotherapy with bladder cancer. Although intraocular sampling did not yield positive results for mycobacterial infection, the patient's ocular disease had responded well to anti-tubercular therapy and topical steroids, which led to a presumed diagnosis of ocular TB from the disseminated BCG infection. In addition to demonstrating a rare ocular complication following BCG treatment, it highlights the difficulties in the diagnosis of ocular TB due to low diagnostic yield and the importance of clinicians having a high index of suspicion to diagnose and treat the disease.

## Introduction

Intravesical Bacillus Calmette-Guérin (BCG) immunotherapy is a commonly utilised adjunct in the treatment of high-risk non-muscle-invasive bladder cancer following transurethral resection of bladder tumour (TURBT). BCG is a live attenuated vaccine form of the bacterium *Mycobacterium bovis*, which infects host uroepithelial cells to trigger an innate immune response. This innate immune response in turn activates macrophages, dendritic cells, and T lymphocytes, which causes an anti-tumour effect on the tumour cells [1].

Common side effects include dysuria, haematuria, and balanitis. Rarely, a traumatic catheterisation can result in a localised tuberculosis (TB) infection "BCG-itis", which manifests as localised ulcerations or granulomatous lesions, regional lymphadenitis, or a disseminated TB infection "BCG-osis", which manifests as fevers, night sweats, weight loss, and distant organ involvement, such as the lungs, spleen, and liver. In rare instances, there is ocular involvement, with tuberculous uveitis (TBU) and granulomatous lesions as potential manifestations [2,3].

This case report presents a patient with presumed intraocular and extraocular TB following BCG immunotherapy, alongside the challenges in diagnosis and treatment.

## Case presentation

The patient is a 64-year-old Caucasian gentleman who was diagnosed with multifocal high-grade, non-invasive bladder cancer. He had initially undergone a TURBT and was commenced on BCG immunotherapy two months post-operatively. He had a past medical history of chronic obstructive pulmonary disease, gastro-oesophageal reflux disease, hypertension, and hypercholesterolemia and denied any past ocular history.

BCG was instilled weekly, with the first two instillations of the BCG treatment being uncomplicated. However, the third instillation resulted in a traumatic catheterisation. The patient had a further fourth and fifth administration of the BCG treatment, each a week apart, before presenting to the emergency department (ED) with severe pain and inflammation of his foreskin prior to the sixth dose, which was subsequently withheld. 

**Figure 1:**
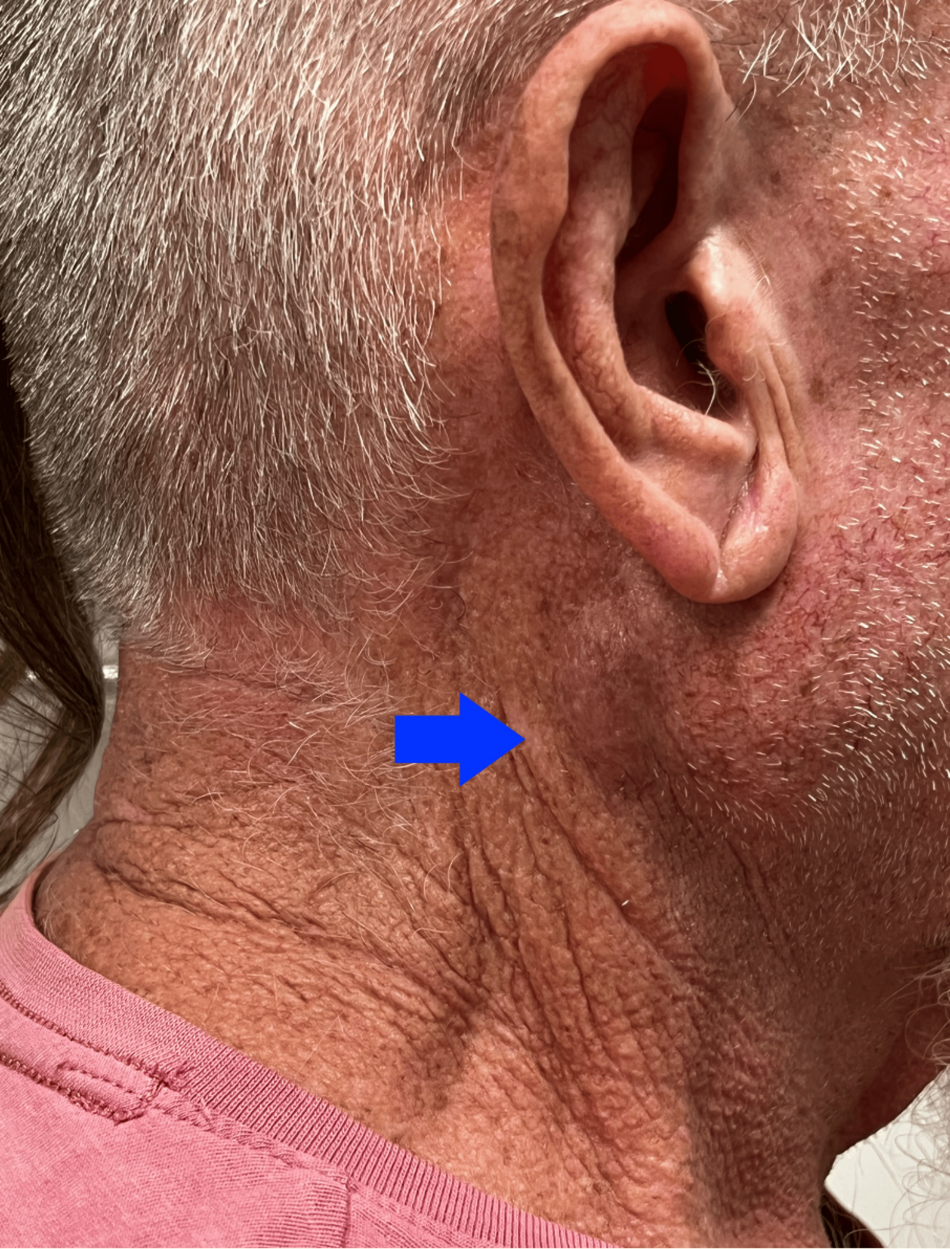
Enlarged parotid node (indicated by a blue arrow)

**Figure 2:**
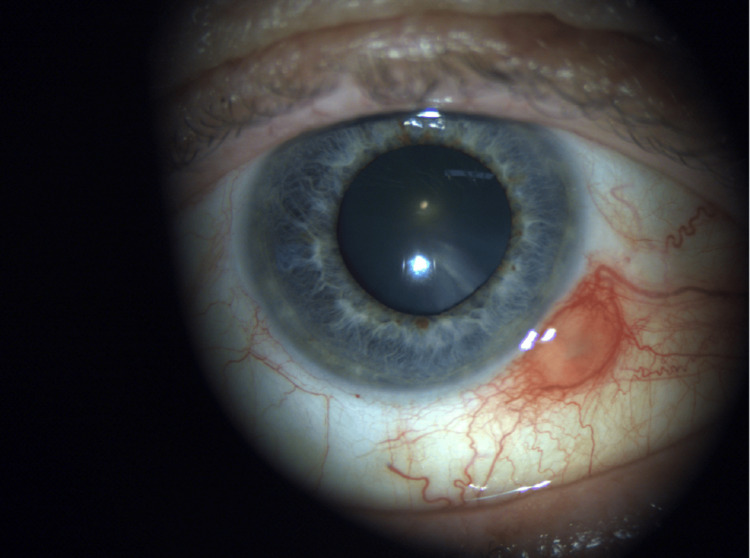
Conjunctival lesion in the patient's right eye at the initial presentation to the ophthalmology department

At the time of presentation to the ED, the patient had also complained of intermittent fevers and night sweats over the last four weeks, with 12 kg of unintentional weight loss. He had also developed an enlarged parotid lymph node (Figure 1). Finally, he also complained of redness and photophobia in his right eye over the last month and had noticed a new conjunctival swelling (Figure 2).

He was referred to the ophthalmology team for the assessment of his red eye and conjunctival lesion. On review, the visual acuity in the affected eye was 6/15 compared to the 6/6 of the unaffected eye. There was a 1×1 mm conjunctival cystic lesion nasal to the cornea, as well as an anterior uveitis with 2+ cells in the anterior chamber (AC) of the eye. The examination of the posterior pole was normal, without any signs of inflammation.

He was admitted under the infectious diseases team and had undergone multiple investigations, including blood tests, computed tomography (CT) imaging of his chest, abdomen, and pelvis, and a parotid core biopsy. At the time of admission, he had also undergone an AC tap of the eye, although the cystic lesion itself was not biopsied or excised on initial review due to concerns of a differential diagnosis of ocular surface squamous neoplasia.

The relevant positive findings included abnormal liver function tests with a cholestatic pattern. Serology for human immunodeficiency virus (HIV), hepatitis B, and hepatitis C was negative. He had positive IgG but negative IgM for the Epstein-Barr virus and cytomegalovirus, indicating past exposure only. The parotid core biopsy was positive for acid-fast bacilli (AFB), and a GeneXpert panel confirmed *Mycobacterium tuberculosis* complex. The AC tap did not yield any positive results, including the GeneXpert panel for *Mycobacterium*.

Due to the systemic findings, the patient was diagnosed with a disseminated TB infection, "BCG-osis", and commenced on anti-tubercular therapy (ATT) during his admission and was treated for a duration of nine months. He was also commenced on corticosteroid eye drops for his anterior uveitis following his ophthalmology review. 

**Figure 3:**
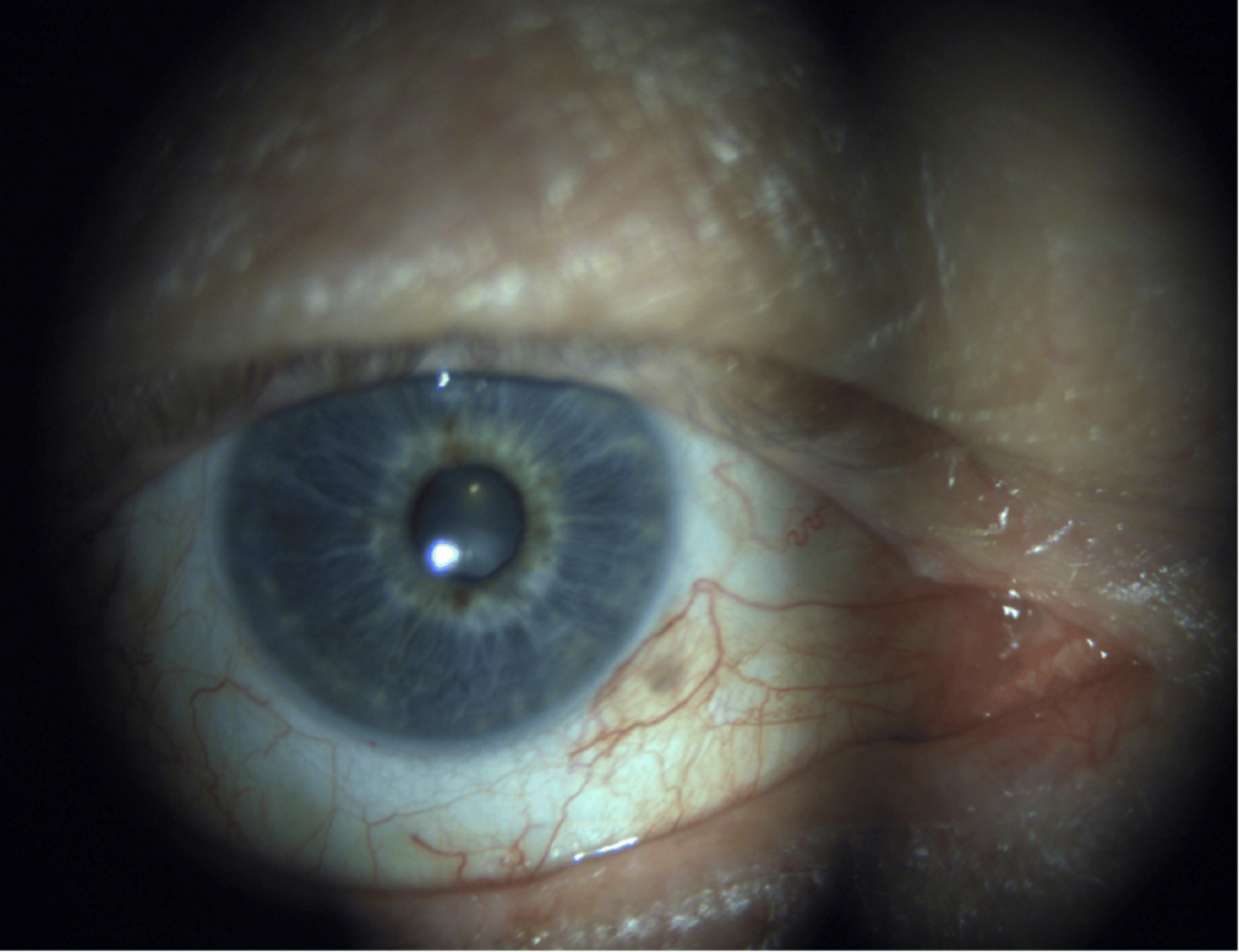
Resolution of conjunctival swelling after the commencement of anti-tubercular therapy and topical corticosteroid eye drops

In terms of the ocular manifestations of his disseminated TB infection, the patient had responded rapidly to the commencement of ATT and topical corticosteroid eye drops. Within a week of follow-up review, the AC inflammation had resolved completely, and the cystic lesion had significantly reduced in size (Figure 3). At the last ophthalmology follow-up at the three-month mark, there were no signs of recurrence of ocular disease.

## Discussion

Ocular complications following BCG immunotherapy are extremely rare and have been estimated in pre-existing literature to be less than 5%. Complications can be divided into extraocular and intraocular manifestations of TB. In the extraocular form, direct inoculation by the mycobacteria or a haematogenous spread can affect the eyelids, lacrimal system, conjunctiva, cornea, and sclera to cause abscesses, dacryoadenitis, phlyctenules, granulomatous tuberculomas, keratoconjunctivitis, and scleritis [4].

Complications from intraocular TB manifest as TBU and optic neuritis. The underlying mechanism of TBU is believed to result from antigenic mimicry, stemming from cross-reactivity between mycobacterial antigens and ocular proteins. The activation of T lymphocytes activates a delayed hypersensitivity reaction within the eye. The optic nerve may be affected by a direct haematogenous spread of mycobacteria or may be affected by the immune response as described above [5].

In this case, it is important to note that the diagnosis of TBU is purely presumptive. Although the AC tap did not yield any positive results, the ophthalmology team had felt that the anterior uveitis and conjunctival lesion were attributable to the disseminated TB infection, given the acute onset of ocular signs and symptoms coinciding with the other systemic features. It was noted that the patient's anterior uveitis and conjunctival lesion had responded well to ATT and topical steroids. The concurrent use of ATT and topical steroids makes it hard to determine which medication was effective at quelling the AC inflammation. However, it is of note that the patient did not develop any rebound ocular inflammation or recurrence of the conjunctival lesion after being weaned from topical steroids whilst he remained on his course of ATT.

Other causes of uveitis were excluded as well, which further pointed to TBU. Another differential for the conjunctival lesion could have been an ocular surface squamous cell neoplasia. There were initial reservations to perform a biopsy at the time of initial review due to concerns of dissemination of potential cancer. This, in conjunction with the resolution of the lesion upon commencement of ATT and steroids, further pointed towards a clinical picture of intra- and extraocular manifestations of TB. 

Additionally, it is also worthwhile noting that intraocular sampling to aid the diagnosis of intraocular TB has low sensitivity rates of approximately 10-40%, relative to the potentially serious complications, such as perforation, hyphaema, and endophthalmitis. As such, most of the published research on TBU are based on a presumptive diagnosis of TBU, aided by a combination of clinical findings and immunological or radiological evidence of TB infection [6,7].

A pooled analysis of ocular complications following BCG immunotherapy for bladder cases had found that the commonest manifestations were conjunctivitis (17 cases), followed by uveitis (seven cases) and endophthalmitis (five cases). As far as the clinical team is aware, this appears to be one of the few reported cases of presumed simultaneous intraocular and extraocular involvement following intravesical BCG therapy [8].

## Conclusions

Ocular involvement in BCG-osis is a rare complication of BCG immunotherapy. Clinicians should be aware that the mycobacteria can induce either intraocular or extraocular manifestations and rarely there may be both intraocular and extraocular manifestations. Additionally, signs and symptoms of ocular involvement may be relatively subtle, which warrants a greater index of suspicion in managing such patients. Finally, the relatively low yield from intraocular sampling often necessitates presumptive diagnosis of ocular TB in conjunction with the clinical history, examination, and immunological or radiological testing.
